# Blood‐based inflammatory protein biomarker panel for the prediction of relapse and severity in patients with neuromyelitis optica spectrum disorder: A prospective cohort study

**DOI:** 10.1111/cns.14811

**Published:** 2024-06-23

**Authors:** Quanfeng Wei, Jiahong Li, Chenyang Zhao, Su Meng, Na Liu, Zhe Wu, Fang Liu, Lingling Cui, Wenyu Hu, Yinan Zhao

**Affiliations:** ^1^ Department of Neurology The First Hospital of China Medical University Shenyang Liaoning China; ^2^ Department of Neurology, Xuanwu Hospital, National Center for Neurological Disorders Capital Medical University Beijing China; ^3^ Department of Radiology The First Hospital of China Medical University Shenyang Liaoning China; ^4^ Department of Cardiology The First Hospital of China Medical University Shenyang Liaoning China

**Keywords:** biomarker panel, neuromyelitis optica spectrum disorders, plasma inflammatory proteins, prognostic model

## Abstract

**Background:**

To date, most existing models for predicting neuromyelitis optica spectrum disorder (NMOSD) are based primarily on clinical characteristics. Blood‐based NMOSD severity and prognostic predictive immune‐ and inflammation‐related biomarkers are needed. We aimed to investigate the associations between plasma inflammatory biomarkers and relapse and attack severity in NMOSD.

**Methods:**

This two‐step, single‐center prospective cohort study included discovery and validation cohorts. We quantified 92 plasma inflammatory proteins by using Olink's proximity extension assay and identified differentially expressed proteins in the relapse group (relapse within 1 year of follow‐up) and severe attack group. To define a new molecular prognostic model, we calculated the risk score of each patient based on the key protein signatures and validated the results in the validation cohort.

**Results:**

The relapse prediction model, including FGF‐23, DNER, GDNF, and SLAMF1, predicted the 1‐year relapse risk. The severe attack prediction model, including PD‐L1 and MCP‐2, predicted the severe clinical attack risk. Both the relapse and severe attack prediction models demonstrated good discriminative ability and high accuracy in the validation cohort.

**Conclusions:**

Our discovered biomarker signature and prediction models may complement current clinical risk stratification approaches. These inflammatory biomarkers could contribute to the discovery of therapeutic interventions and prevent NMOSD progression.

## Graphical abstract

We quantified 92 inflammatory proteins to screen out key different proteins of NMOSD, established prediction models and validated them in a separate validation group.

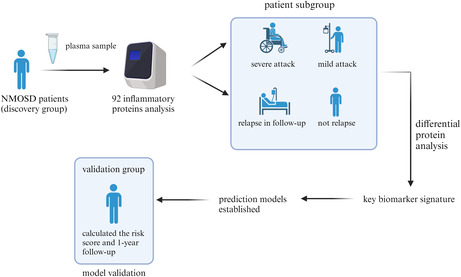

## 1 INTRODUCTION

Neuromyelitis optica spectrum disorder (NMOSD) is an autoimmune disease that affects the central nervous system (CNS) and is characterized by optic neuritis (ON) and transverse myelitis (TM).^1^ Recurrent episodes of these symptoms can lead to disability and decreased quality of life.^2^ Pathogenic aquaporin‐4 water channel autoantibodies (AQP4‐IgG) are believed to be the main cause of this disease.^3^

AQP4‐IgG is now a crucial biomarker for diagnosing NMOSD. However, the presence or level of AQP4‐IgG does not correlate with clinical illness features.^4^ In recent years, numerous biomarkers have been used to monitor and classify disease progression and predict treatment outcomes.^5^ Glial fibrillary acidic protein (GFAP) and S100β are the signature intermediate filament markers of astrocytes and reflect the degree of astrocyte damage in the CNS,^6^, ^7^ and neurofilament light chain (NfL) is an indicator of damage to nerve fibers^8^; however, peripheral blood immune status markers are lacking.

The novel biomarkers that have been suggested to predict the prognosis of NMOSD include IL‐6 and other Th17‐mediated biomarkers, such as IL‐17.^9^, ^10^ The serum or cerebrospinal fluid (CSF) levels of these proinflammatory factors can reflect the intensity of the inflammatory response. IL‐6 might be a useful marker for differential diagnosis. Th17 cells and Th17‐related mediators are believed to be crucial in the immunopathogenesis of NMOSD.^11^ The abovementioned biomarkers are easily accessible, noninvasive, and blood‐based markers for the diagnosis and monitoring of disease progression in patients with NMOSD. Considering that the changes in these markers during NMOSD progression are relatively small, it is difficult for only these markers to clearly and accurately reflect the current disease status or even predict disease prognosis. Moreover, although some markers can indirectly reflect the degree of nerve damage, they lack sufficient predictive efficacy for NMOSD,^12^ and it remains unclear whether those biomarkers can completely characterize the entire underlying pathological mechanism of NMOSD. Therefore, a more comprehensive analysis of protein profiles is needed to determine disease status, patient prognosis, and disease pathways. In a recent study, sophisticated ultrasensitive and high‐throughput protein measurement techniques were utilized to simultaneously measure the expression levels of hundreds of proteins in Alzheimer's disease (AD),^13^ which consequently led to the identification of novel biomarkers and the definition of disease stages. At present, studies on relevant clinical prediction models focusing on the immunoinflammatory features of neuromyelitis spectrum diseases are very limited. Most clinical prediction models are mainly composed of clinical features,^14^, ^15^ including patients' immune features, and lack tools to predict the prognosis and risk of disease progression and recurrence. Existing clinical prediction models have generally been developed based on retrospective data, have performed poorly in assessing disease prognosis, and have several limitations. Therefore, prospective studies on the immunological characteristics of NMOSD are needed.

In this study, we aimed to use the proximity extension assay (PEA) in a prospective cohort to identify novel potential biomarkers and assess their predictive power. Moreover, we created two prognostic models by using a biomarker panel to increase prognostic accuracy and confirm the effectiveness of the model in a separate validation cohort. Thus, we obtained a more comprehensive overview of the acute‐phase plasma proteins of NMOSD and developed a more precise plasma biomarker prediction model to reflect the immunological characteristics of NMOSD.

## 2 METHOD

### 2.1 Study design

This study was designed as a two‐step, prospective, single‐center cohort study of patients with NMOSD. To identify the critical elements that might influence the inflammatory response in NMOSD, we categorized the patients in the discovery cohort into distinct groups according to their clinical characteristics and examined how their inflammatory protein profiles differed from one another. A plasma inflammatory protein prediction model was constructed using these essential components. Subsequently, other patients were assigned to a validation cohort, and these patients were categorized based on the predicted outcomes. The validation set was used to evaluate the performance of the innovative prediction model.

### 2.2 Participant recruitment

The 2015 International Panel on Neuromyelitis Optica Diagnosis criteria served as the foundation for the clinical diagnosis of NMOSD. The exclusion criteria were (1) AQP4‐IgG negativity, (2) age under 16 years, (3) any notable neurological condition other than NMOSD or a psychiatric issue, and (4) lack of sufficient follow‐up or clinical data.

Newly developed or deteriorating neurological symptoms lasting for more than 24 h with objective signs confirmed on neurological examination, with signs and symptoms attributable exclusively to NMOSD rather than other causes, and attack within 30 days were considered the same attack and were the criteria used to define relapse. An experienced neurologist used the Expanded Disability Status Scale (EDSS) at the first clinical episode following enrollment to determine the baseline EDSS score.

Patients were categorized into recurrence and nonrecurrence groups based on the relapse stage and into severe attack (EDSS ≥ 6) and moderate/mild attack groups (EDSS = 0–5.5) based on the EDSS score of the initial attack.

### 2.3 Blood sampling and plasma protein collection and measurement

Blood samples were collected from acute‐phase patients within 24 h of admission and before immunotherapy. Blood samples were collected in EDTA‐containing tubes (BD Vacutainer# 367955). Prior to detection, the serum samples were stored at −80°C following centrifugation at 3000 rpm for 15 min at 4°C. Using PEA, Olink® Proteomics was used to measure the levels of inflammatory proteins in 20 μL of plasma. The Olink Target 96 Inflammation Panel was specifically utilized. Normalized protein expression (NPX) values represent protein expression levels on a log_2_ scale and were used to express the tested plasma protein expression levels.

### 2.4 Differential expression analysis and functional enrichment analysis

We first normalized the proteomic data before performing the data analysis. The data are given as NPX, a relative quantitative unit in log_2_ scale that is produced by preprocessing normalization using NPX Signature software and derived from Ct values of quantitative polymerase chain reaction qPCR. Statistical analysis was conducted with the rstatix package. The data were first examined for variance homogeneity with Levene's test and for a normal distribution with the Shapiro–Wilk test. An unpaired *t*‐test was used if the data were homogeneous and had equal variances, a Welch's *t*‐test was used if the data had a normal distribution but unequal variances, and a nonparametric Wilcoxon rank sum test was used if no normality assumption was met. The screening criterion for differentially expressed proteins was set at *p* < 0.05. The Gene Ontology (GO) and Kyoto Encyclopedia of Genes and Genomes (KEGG) databases served as the foundation for the gene‐function annotation process.

### 2.5 Plasma proteome–NMOSD association analysis and the construction of the predictive model

In this study, we created two prediction models for different clinical outcomes. The possible predictors for the predictive model were selected from among the differentially expressed proteins. Only variables with a *p*‐value less than 0.05 were included in the final prediction model during the univariable analysis. Then, using the expression (NPX) values of each predictor multiplied by its logistic regression coefficient (*β*), we created a prediction model, which can be represented by the following equation: *Y* = (*β*_1_) × (*P*_1_) + (*β*_2_) × (*P*_2_) + …, where *P* is the relative quantification of the protein and *β* is the regression coefficient derived from univariate analysis. The *Y* value was the sum of the expression of each biomarker multiplied by its correlation coefficient. Patients were subsequently categorized into low‐ and high‐risk groups using the optimal cutoff value determined by the model.

### 2.6 Statistical analysis and data visualization

For the statistical analysis, SPSS 24.0 (SPSS, Chicago, IL) was used. Categorical data are shown in groups, whereas continuous data are shown as the median and interquartile range (IQR). The Mann–Whitney *U* or unpaired *t*‐test was used to compare the two groups. Regression analysis was employed to evaluate the relevance of the connections between plasma protein expression levels and clinical outcomes. Receiver operating characteristic (ROC) curve analysis was used to determine the predictive value of the parameters. By using the log‐rank test in Kaplan–Meier survival analysis, relapse trends were determined. The performance of the final models was evaluated in a separate validation cohort. *p*‐values less than 0.05 were considered to indicate statistical significance.

R (4.0.3; http://www.r‐project.org/) was utilized for all bioinformatics studies. R generated the heatmap and volcano plot. The remaining statistical graphics were created with GraphPad Prism v8.0.

## 3 RESULTS

### 3.1 Clinical characteristics of NMOSD patients in both the discovery cohort and the validation cohort

The studies included 50 NMOSD patients who attended the neurology department of The First Affiliated Hospital of China Medical University between July 1, 2020, and June 30, 2023, as well as after 1 year of follow‐up. The baseline clinical and demographic characteristics of the two cohorts are outlined in Table 1.

**TABLE 1:** Baseline demographic and clinical characteristics of the NMOSD patients.

	Discovery cohort	Validation cohort	*p‐*value
Patient number, No.	30	20	
Female sex, No. (%)	30 (100)	19 (95)	>0.999
Age, years, mean ± SD	47.00 ± 15.06	48.25 ± 15.43	0.777
Duration, months (IQR)	48.50 (24.25–92.50)	43.00 (22.25–68.75)	0.685
Hypertension, No. (%)	4 (13.33)	0 (0)	0.140
Diabetes, No. (%)	7 (23.33)	2 (10)	0.285
Coronary heart disease, No. (%)	1 (3.33)	0 (0)	>0.999
Autoimmune diseases, No. (%)	10 (33.33)	7 (35)	0.903
Abnormal rheumatic antibodies, No. (%)	20 (66.67)	10 (50)	0.239
Clinical manifestation, No. (%)			0.481
ON	8 (26.56)	9 (45)	
TM	17 (56.67)	7 (35)	
ST	1 (3.33)	1 (5)	
MIX	4 (13.33)	3 (15)	
EDSS score, median (range)	3 (2–8)	4 (1–7)	0.108
Number of relapsed patients, *n* (%)	10 (33.33)	9 (45)	0.405
Time from symptom onset to second attack, months, median (range)	6.50 (4–12)	6 (4–12)	0.681
Acute attack therapy, No. (%)			
IVMP	28 (93.33)	19 (95)	>0.999
IVIG	1 (3.33)	1 (5)	>0.999
PE	1 (3.33)	0 (0)	>0.999
Maintenance therapy, No. (%)			
Prednisone (>6 months)	20 (66.67)	18 (90)	0.058
MMF	11 (36.67)	10 (50)	0.349
TAC	1 (3.33)	1 (5)	>0.999
RTX	9 (30)	2 (10)	0.094
CTX	1 (3.33)	0 (0)	>0.999
Telitacicept	1 (3.33)	0 (0)	>0.999

*Note*: Numerical data are presented as the *n* (%), mean ± SD, or median (IQR).

Abbreviations: CTX, cyclophosphamide; EDSS, Expanded Disability Status Scale; IVIG, intravenous immunoglobulin; IVMP, intravenous methylprednisolone; MIX, mixed attack; MMF, mycophenolate mofetil; NMOSD, neuromyelitis optica spectrum disorder; ON, optic neuritis; PE, plasma exchange; RTX, rituximab; ST, brainstem syndrome; TAC, tacrolimus; TM, transverse myelitis.

The mean age, female proportion, and median duration were not significantly different between the discovery and validation cohorts (age: mean ± standard deviation [SD], 47.00 ± 15.06 vs. 48.25 ± 15.43 years, *p* = 0.777; female sex (%): 100% vs. 95%, *p* > 0.999; duration: 48.50 vs. 43.00 months, *p* = 0.685). In addition, the most common coexisting disease was autoimmune disease, and the most common symptoms were TM and ON, with no difference in incidence in either cohort. Moreover, the EDSS score and time from symptom onset to second attack at the time of serum sampling were comparable between the cohorts (median ± range: 3 (2–8) vs. 4 (1–7), *p* = 0.108; 6.50 (4–11) vs. 6 (4–12), *p* = 0.681). Intravenous methylprednisolone (IVMP) was the most commonly used treatment for acute attacks, and prednisone and mycophenolate mofetil (MMF) were the most frequently used maintenance therapies in both cohorts. Among those who were followed up in our study, 19 patients experienced relapse in the discovery and validation cohorts, and the overall relapse rate was 38%. The details of the clinical and demographic characteristics of the relapsed and nonrelapsed patients are presented in Table S1. In the validation cohort, patients who experienced relapse had significantly higher EDSS scores than patients who did not relapse (*p* = 0.019).

### 3.2 Identification of differentially expressed inflammatory proteins in the relapse and attack severity groups with NMOSD

**FIGURE 1:**
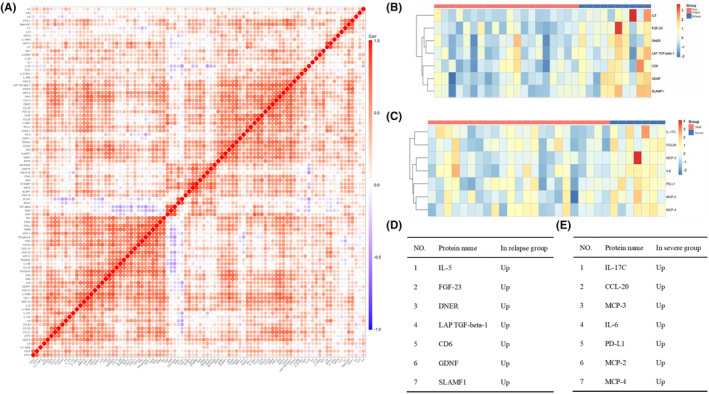
Identification of differentially expressed proteins and their comparison between different groups. (A) Heatmap showing the Spearman correlation coefficient for the inflammatory protein panel. Each row and column represents 1 of the 92 inflammatory‐associated plasma proteins. Red and blue indicate positive and negative correlations between protein pairs, respectively. (B, C) Clustering heatmap of differentially expressed inflammatory proteins in different groups of patients with neuromyelitis optica spectrum disorder. (D, E) Differentially expressed proteins identified in each group.

**FIGURE 2:**
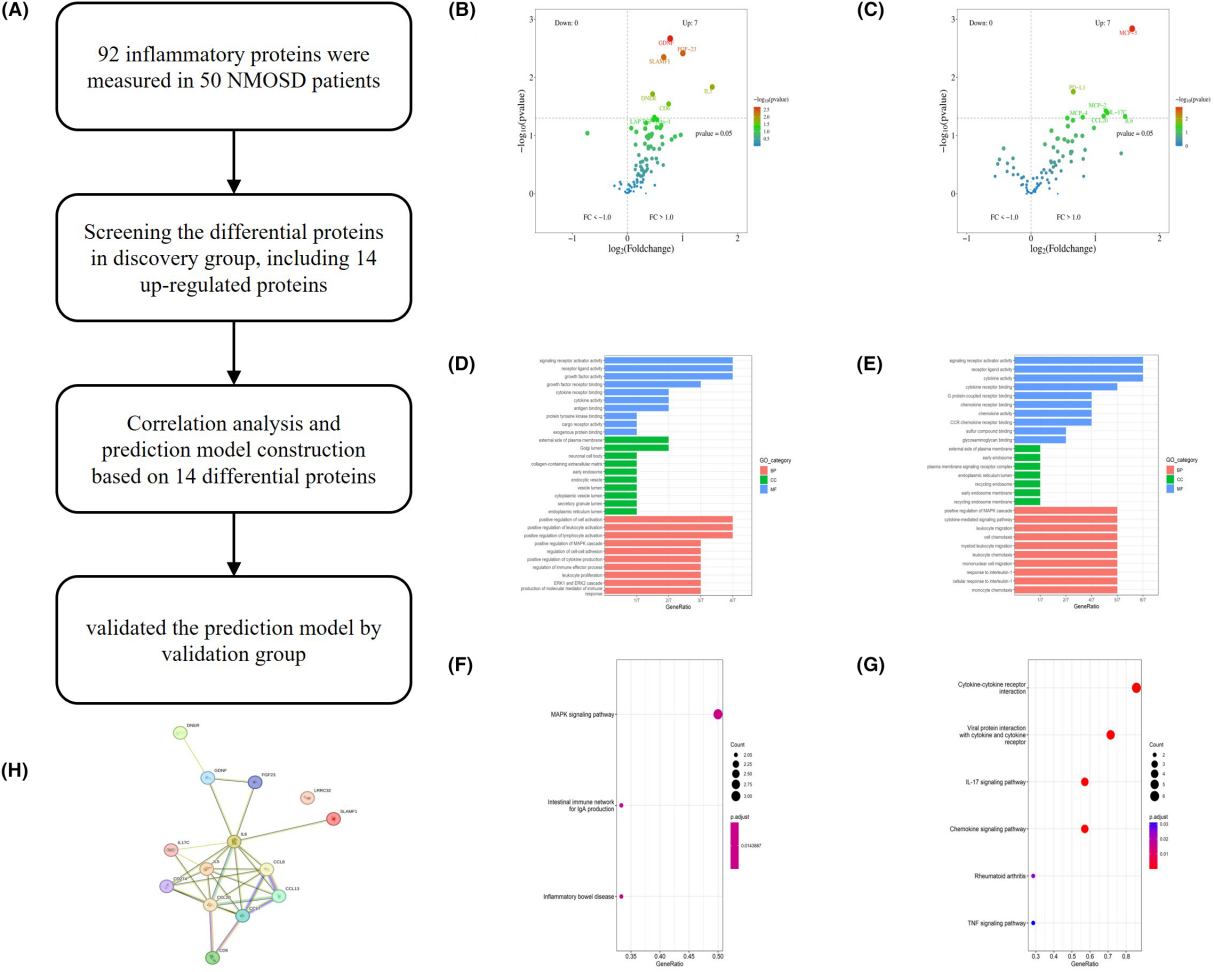
Further analyses of DEPs. (A) Pipeline for the establishment and evaluation of the molecular prognostic model. (B, C) Volcano plot showing the distribution of upregulated and downregulated DEPs in the relapse group (B) and severe attack group (C). (D, E) GO terms of the DEPs in the relapse group (D) and severe attack group (E). (F, G) KEGG pathway analysis of the DEPs in the relapse group (F) and severe attack group (G). (H) Mutual protein interactions among the 14 DEPs. DEPs, differentially expressed proteins; GO, Gene Ontology; KEGG, Kyoto Encyclopedia of Genes and Genomes; NMOSD, neuromyelitis optica spectrum disorder.

We first determined the correlations among the expression levels of 92 inflammatory proteins by Spearman's coefficient correlation analysis. In the heatmap, red represents a positive correlation between two proteins, blue represents a negative correlation, and white represents no significant correlation (Figure 1A). Quantitative analysis of the different groups revealed that the expression levels of seven proteins were significantly different between the relapse group and the nonrelapse group, and the expression levels of seven other proteins were significantly different between the severe attack group and the mild attack group (false discovery rate [FDR]‐adjusted *p* < 0.05). The hierarchical clustering of differentially expressed proteins is shown in the heatmap (Figure 1B,C). Clustering heatmaps and volcano plots revealed that all the differentially expressed proteins tended to be upregulated in the relapse group and severe attack group (Figures 1D,E and 2B,C). These proteins have all been reported to be related to the occurrence and development of neuroinflammatory diseases and the proliferation of various immune cells.^16^, ^17^, ^18^, ^19^, ^20^, ^21^, ^22^, ^23^, ^24^, ^25^, ^26^, ^27^

### 3.3 Results of bioinformatics analysis of identified differentially expressed proteins

The study procedure flow chart is shown in Figure 2A. Proteins exhibiting significant changes (*p* < 0.05) are shown on the volcano map (Figure 2B,C). We conducted GO functional enrichment analysis and KEGG pathway enrichment analysis on 14 screened differentially expressed proteins. GO functional analysis revealed that both relapse‐associated and severity‐associated proteins were mostly related to several molecular functions (MFs), including signaling receptor activator activity, receptor ligand activity, cytokine receptor binding, and cytokine activity. GO cellular component (CC) analysis terms mainly included the external side of the plasma membrane and early endosome. The GO biological process (BP) analysis terms mainly included positive regulation of the MAPK cascade (Figure 2D,E). According to KEGG analysis, relapse‐related differentially expressed proteins were mainly enriched in the MAPL signaling pathway, the intestinal immune network for IgA production and inflammatory bowel disease; the severity‐related differentially expressed proteins were mainly enriched in cytokine–cytokine receptor interaction, the IL‐17 signaling pathway and the chemokine signaling pathway (Figure 2F,G). Finally, we constructed protein–protein interaction networks (Figure 2G) using the STRING database to better understand the interactions of these proteins.

### 3.4 Identifying key plasma proteins associated with the clinical manifestations of NMOSD

**FIGURE 3:**
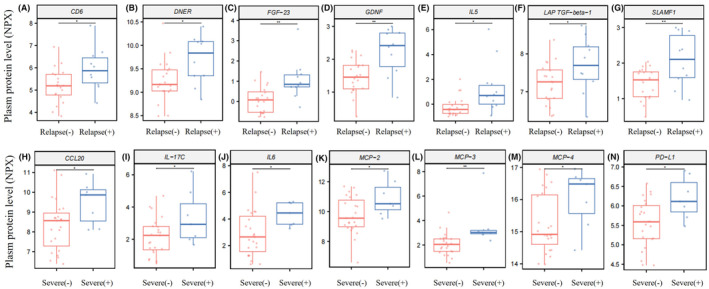
Quantitative analysis of plasma proteins in different groups. (A–G) Box plots illustrating different plasma levels of CD6 (A), DNER (B), FGF‐23 (C), GDNF (D), IL5 (E), LAP TGF‐beta‐1 (F), and SLAMF1 (G) in the relapse group (*n* = 10) and nonrelapse group (*n* = 20). (H–N) Different plasma levels of CCL20 (H), IL‐17C (I), IL6 (J), MCP‐2 (K), MCP‐3 (L), MCP‐4 (M), and PD‐L1 (N) in the severe attack group (*n* = 7) and mild/moderate attack group (*n* = 23). *p < 0.05, **p < 0.01.

The expression levels of 14 significantly differentially expressed proteins are shown in Figure 3. To determine the key proteins, we performed a univariable analysis on the screened differentially expressed proteins. Except for LAP TGF‐beta‐1, most differentially expressed proteins found in the relapse group correlated positively with NMOSD relapse. FGF‐23, DNER, GDNF, and SLAMF1 were significantly correlated (*p* < 0.05) with both relapse and relapse numbers. PD‐L1 and MCP‐2 were significantly correlated (*p* < 0.05) with both severe attack and EDSS scores at disease onset. All differentially expressed proteins found in the severe group exhibited significant correlations with EDSS scores (Table 2).

**TABLE 2:** The coefficients and *p* values of different factors in the univariable logistic regression.

	Relapse		Relapse number		Severe attack		EDSS	
	Coefficient	*p* value	Coefficient	*p* value	Coefficient	*p* value	Coefficient	*p* value
IL‐5	0.938	0.047*	0.073	0.418	−0.347	0.419	−0.07	0.757
FGF‐23	1.893	0.017*	0.573	0.05*	0.48	0.029	0.25	0.513
DNER	1.994	0.032*	0.48	0.029*	0.374	0.658	0.605	0.371
LAP TGF‐beta‐1	1.339	0.062*	0.3	0.09	0.777	0.27	0.683	0.203
CD6	1.104	0.048*	0.235	0.07	−0.072	0.885	−0.083	0.832
GDNF	2.193	0.01*	0.288	0.04*	0.516	0.426	0.511	0.31
SLAMF1	2.154	0.019*	0.37	0.024*	0.448	0.516	0.523	0.347
IL‐17C	0.014	0.963	0.015	0.865	0.664	0.062	0.631	0.009**
CCL‐20	0.102	0.732	0.059	0.505	0.741	0.061	0.66	0.08*
MCP‐3	0.184	0.524	0.158	0.065	1.388	0.055	0.585	0.019*
IL‐6	−0.083	0.719	0.01	0.886	0.557	0.066	0.407	0.038*
PD‐L1	0.482	0.438	0.196	0.272	2.107	0.035*	1.303	0.01*
MCP‐2	0.533	0.123	0.155	0.038*	0.852	0.05*	0.56	0.028*
MCP‐4	0.593	0.164	0.218	0.066	0.955	0.066	0.673	0.055

Abbreviation: EDSS, Expanded Disability Status Scale.

*p < 0.05, **p < 0.01.

**FIGURE 4:**
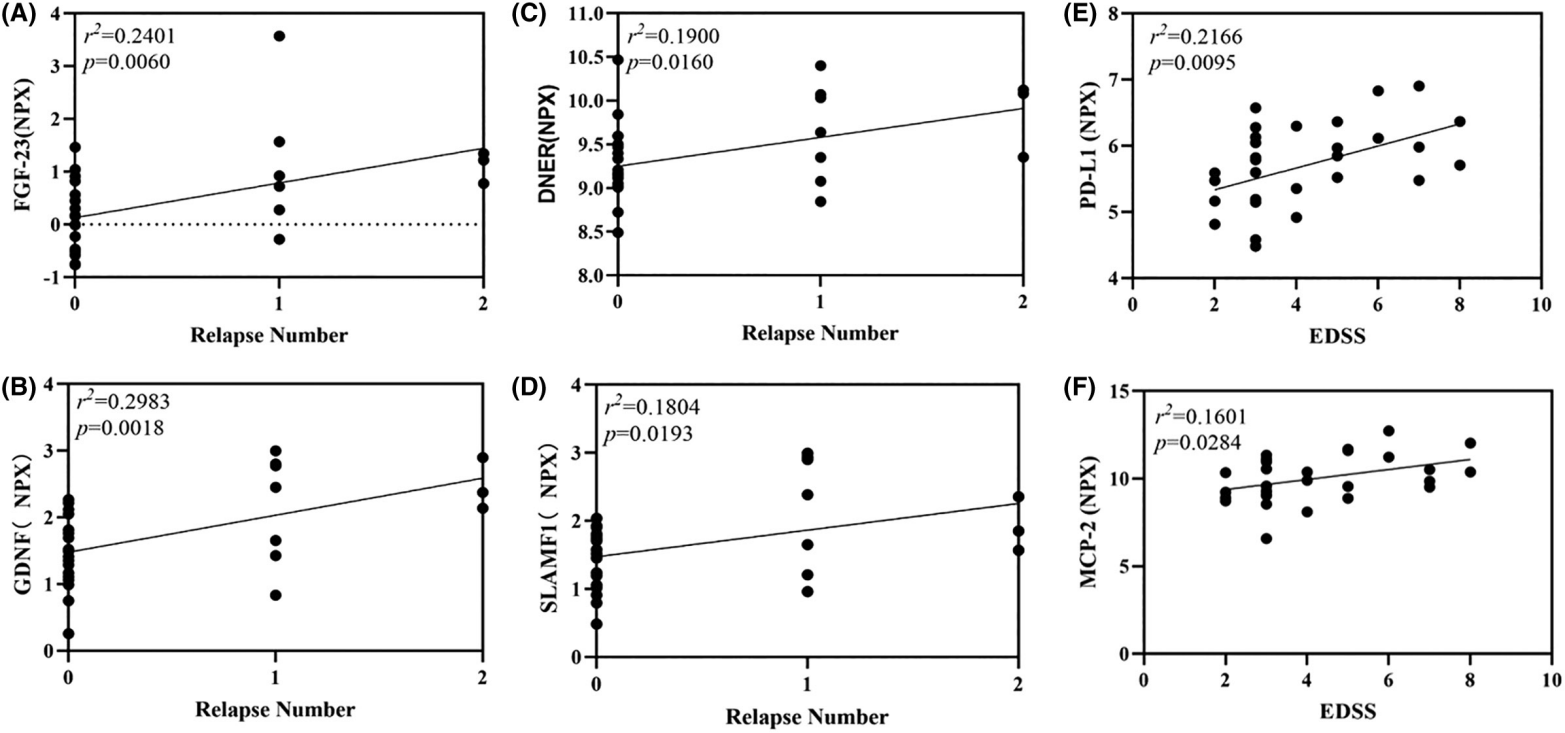
Correlations between the plasma levels of differentially expressed proteins and clinical parameters. (A–D) Scatter plots representing the associations between the plasma levels of the relapse‐related proteins FGF23, DNER, GDNF, and SLAMF1 and the number of relapses. FGF‐23 (*r* ^2^ = 0.2401, *p* = 0.0060, 95% CI = 0.2051–1.112), DNER (*r* ^2^ = 0.1900, *p* = 0.0160, 95% CI = 0.06636–0.5949), GDNF (*r* ^2^ = 0.2983, *p* = 0.0018, 95% CI = 0.2256–0.8850), and SLAMF1 (*r* ^2^ = 0.1804, *p* = 0.0193, 95% CI = 0.06846–0.7146). (E, F) Scatter plots representing the association between the plasma levels of the severity‐related proteins MCP‐2 and PD‐L1 and the EDSS score. MCP‐2 (*r* ^2^ = 0.1601, *p* = 0.0284, 95% CI = 0.03245–0.5393), PD‐L1 (*r* ^2^ = 0.2166, *p* = 0.0095, 95% CI = 0.04385–0.2885). CI, confidence interval; EDSS, Expanded Disability Status Scale; *r* ^2^, the square of Pearson's correlation coefficient.

Next, we constructed a scatter plot to demonstrate the relationships between key plasma proteins and clinical manifestations. Among the relapse‐related proteins, plasma FGF‐23 (*r* ^2^ = 0.2401, *p* = 0.0060), DNER (*r* ^2^ = 0.1900, *p* = 0.0160), GDNF (*r* ^2^ = 0.2983, *p* = 0.0018), and SLAMF1 (*r* ^2^ = 0.1804, *p* = 0.0193) were significantly positively correlated with relapse number (Figure 4A–D). A similar trend was found for severity‐related proteins: the plasma levels of PD‐L1 and MCP‐2 were significantly positively correlated with the EDSS score (MCP‐2 (*r* ^2^ = 0.1601, *p* = 0.0284) and PD‐L1 (*r* ^2^ = 0.2166, *p* = 0.009)) (Figure 4E,F).

### 3.5 Validation of two molecular prognostic models based on key differentially expressed proteins in the validation cohort

We created two efficient prediction models based on a protein biomarker panel. Significant proteins in the univariable logistic regression analysis were included in the panel.

**FIGURE 5:**
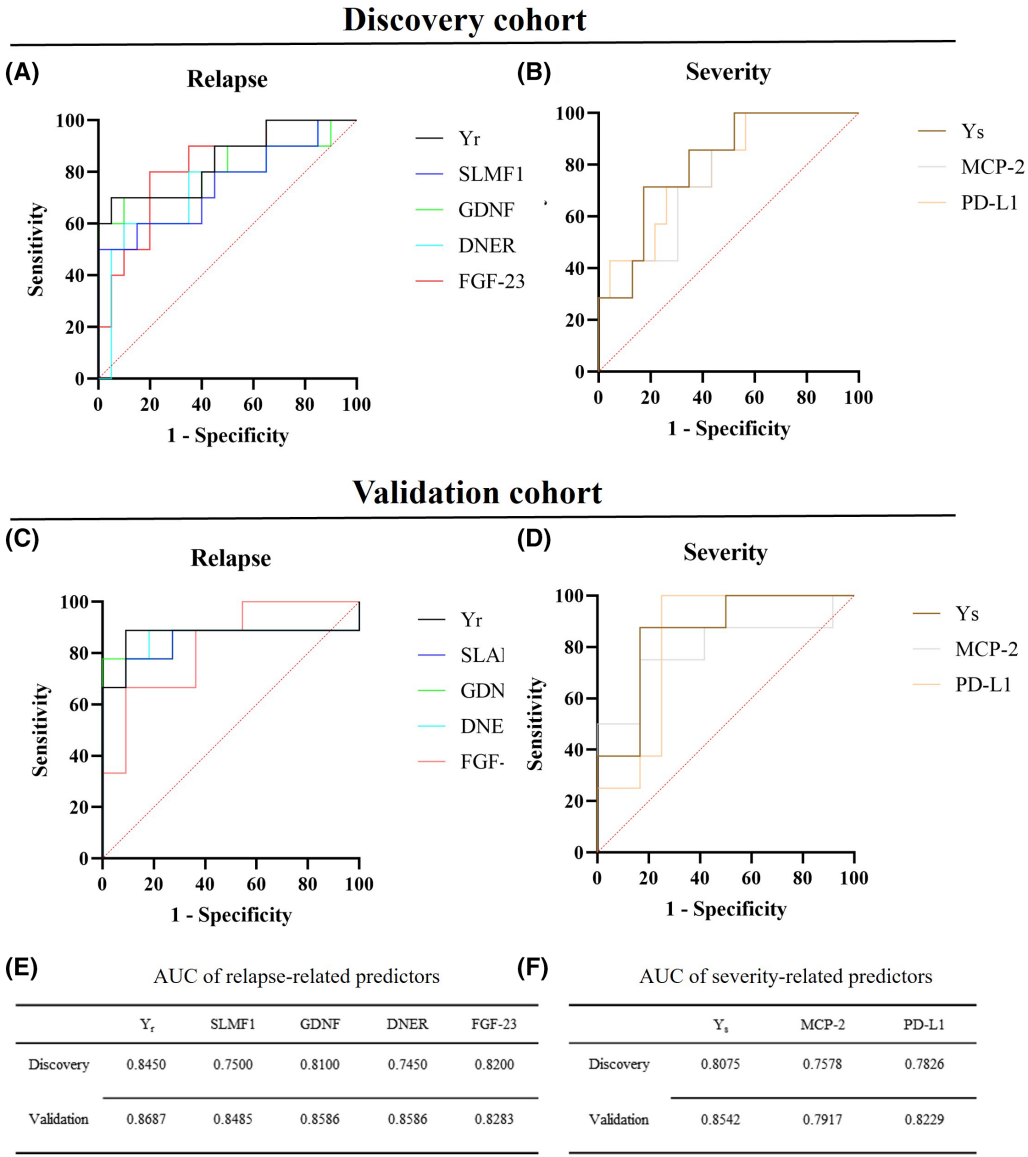
ROC curve showing the specificity and sensitivity of key proteins and the prognostic model for clinical relapse and severity. (A, B) ROC curve for biomarkers and prognostic model indicating specificity and sensitivity to discriminate patients with relapse and severe attack from the control group in the discovery cohort. (C, D) ROC curve for biomarkers indicating specificity and sensitivity to discriminate patients with relapse and severe attack from the control group in the validation group. (E, F) AUCs of the relapse‐related predictors and severity‐related predictors in the discovery and validation cohorts. AUC, area under the ROC curve; CI, confidence interval; ROC, receiver operating characteristic; *Y*_r_, prognostic model for relapse; *Y*_s_, prognostic model for severe attack.

The equation for the risk of relapse model was *Y*_r_ = 0.573(FGF‐23) + 0.48(DNER) + 0.288(GDNF) + 0.37(SLAMF1), and the equation for the risk of severe disease model was *Y*_s_ = 2.107(PD‐L1) + 0.852(MCP2). Figure 5A–D shows the ROC curves of the predictive model and each key protein in the discovery cohort and the validation cohort. Both the model and the single key protein had good discriminatory capacity. Figure 5E,F shows the area under the ROC curve (AUC) values for each predictor in the ROC curve analysis. After calculating the AUC values of the different predictors, we found that the two predictive models had the highest AUC values in both the discovery and validation cohorts (*Y*_r_ [discovery: 0.8450 (95% CI: 0.6842–1); validation: 0.8687 (95% CI: 0.6617–1)]; *Y*_s_ [discovery: 0.8075 (95% CI: 0.6391–0.9758); validation: 0.8542 (95% CI 0.6832–1)]) compared with the single key protein. In addition, we combined more differentially expressed single proteins and performed ROC curve analysis, and all combinations had high predictive values (AUC > 0.7, Table S2).

**FIGURE 6:**
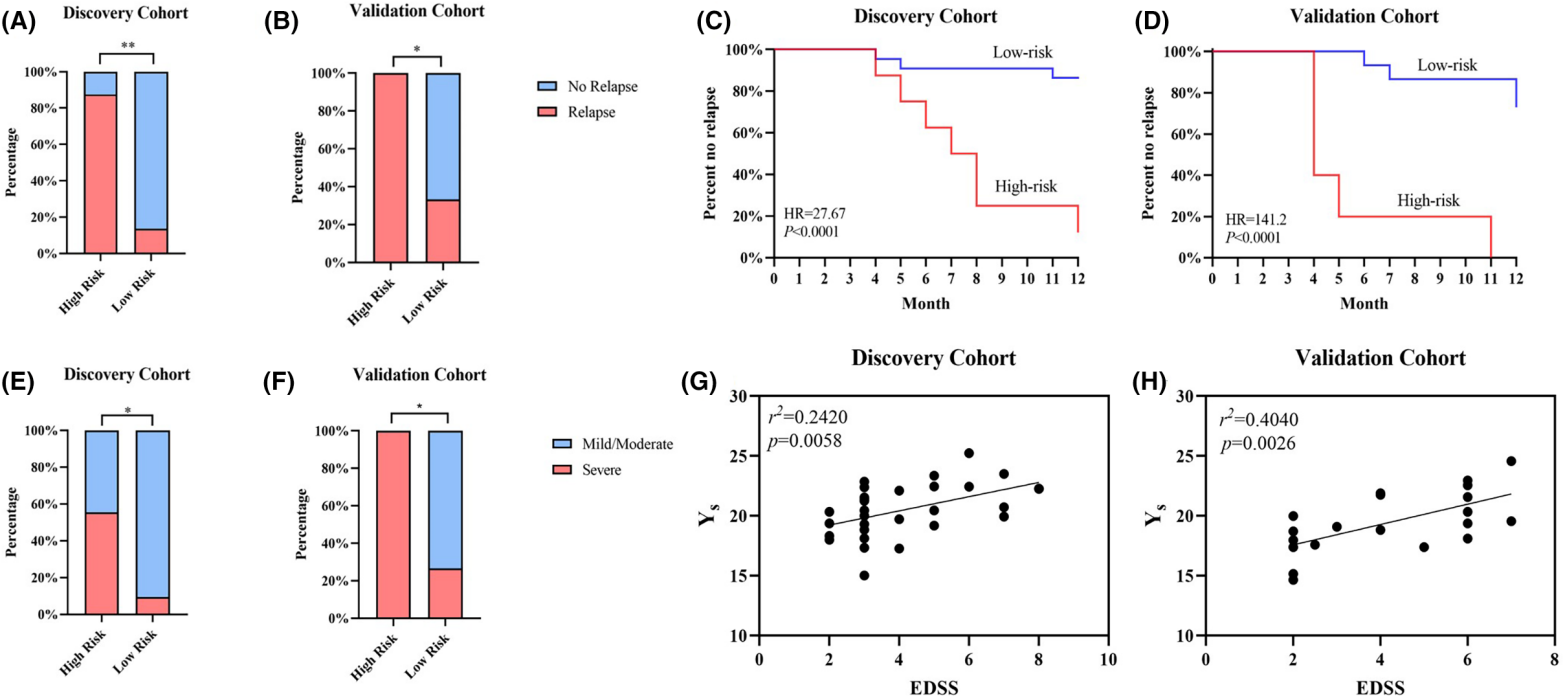
Identification of two novel molecular prognostic models in NMOSD patients. (A, B) Stacked column charts showing the proportion of patients who experienced relapse in different groups. Patients were divided into low‐ and high‐risk groups based on the Youden index of the relapse hazard model. (C, D) Kaplan–Meier plots showing that patients in the low‐ and high‐risk groups had contrasting relapse rates. (E, F) Stacked column charts showing the proportion of patients with severe attacks in different groups. Patients were divided into low‐ and high‐risk groups based on the Youden index of the severe attack hazard model. (G, H) Correlation between the *Y*_s_ score and EDSS score. *Y*_s_ was positively correlated with the EDSS score (Discovery cohort: *r* ^2^ = 0.2420, *p* = 0.0058; validation cohort: *r* ^2^ = 04040, *p* = 0.0026). EDSS, Expanded Disability Status Scale; NMOSD, neuromyelitis optica spectrum disorder; *Y*_s_, prognostic model for severe attack; *p < 0.05, **p < 0.01.

*Y* scores were used to divide patients into high‐risk and low‐risk categories. The optimal cutoff value was determined by calculating the Youden index (*Y*_r_: 6.4987; *Y*_s_: 22.1886). In the discovery cohort, 87.5% of patients in the high‐risk group experienced relapse and 13.63% of patients in the low‐risk group experienced relapse. The severe attack risk group had a 55.56% severe attack rate, and the low‐risk group had a 9.52% severe attack rate. The validation cohort showed similar results (high relapse risk group: 100%; low relapse risk group: 26.67%; high severe attack risk group: 100%; low severe attack group: 29.41%; Figure 6A,B,E,F). Kaplan–Meier analysis confirmed that patients in the high relapse risk group experienced a greater relapse rate (discovery cohort: *p* < 0.0001; validation cohort: *p* < 0.0001; Figure 6C,D). The scatter plot and linear fit plot show the relationship between the *Y*_s_ score and the EDSS score (discovery cohort: *r* ^2^ = 0.2420, *p* = 0.0058; validation cohort: *r* ^2^ = 0.4040, *p* = 0.0026; Figure 6G,H).

## 4 DISCUSSION

NMOSD is characterized by high relapse and disability rates, but the application of immune/inflammatory protein signatures for predicting patient prognosis is still rare. In our study, the plasma inflammatory protein profile during acute illness in NMOSD patients was analyzed, and we identified six key NMOSD‐associated plasma proteins. Next, we explored their clinical value and developed and validated two prediction models in a prospective validation cohort. The most important finding of this study is that a differential inflammatory protein biomarker panel in blood can be used clinically to predict severity and relapse risk with greater sensitivity and specificity than single biomarkers. This panel can help us understand the immune‐mediated inflammatory pathways underlying disease, and the proteins in this panel can serve as targets for novel treatment approaches. The model can be used to differentiate patients and manage their disease more accurately, and the model performs well in predictions. Simultaneously, as peripheral blood samples are readily available, the present cost of monitoring the disease course status of NMOSD patients (imaging, cerebrospinal fluid indicators) can be reduced; thus, this model has good prospects for clinical transformation and application.

There are scientific concerns about the role that immune homeostasis instability plays in the onset of autoimmune diseases. Scientists are still learning about how the intricate balancing act of the immune system is managed and how it occasionally goes wrong due to the COVID‐19 pandemic.^28^ AQP4‐IgG can cause complement‐dependent cytotoxicity (CDC), which disrupts the blood–brain barrier and results in the recruitment of inflammatory immune cells and the release of inflammatory cytokines. Additionally, AQP4‐IgG can cause a variety of secondary oligodendrocyte injuries, demyelination, and neuronal injuries,^29^, ^30^ thereby defining NMOSD as a complex inflammatory response.

To accommodate the fluctuation of plasma levels at different sampling times, an inflammatory factor model was used rather than a single marker for distinguishing the severity and relapse risk of NMOSD. One benefit of this NMOSD inflammatory factor model is that it provides a comprehensive, precise, and targeted approach to studying the pathogenesis of various diseases. The model offers significant advantages in terms of multiplex analysis, sensitivity, specificity, and scalability, which may result in improved therapeutic and diagnostic strategies as well as a deeper understanding of the relevant inflammatory processes. The proposed model was able to predict the clinical course at different ends of the heterogeneous disease spectrum of NMOSD, and it was able to identify both NMOSD patients with low and high probabilities of achieving a high EDSS score and patients with relapse risk in our study with a replication cohort. Given that early intervention has been shown to improve disability outcomes over the long term, even in patients in whom the disease initially appears to be mild,^31^ a biomarker that indicates a greater likelihood of enduring long‐term disability advancement and can be identified during the initial phases of the illness would support the early diagnosis of NMOSD and prompt high‐efficacy treatment. The protein biomarkers identified showed great predictive potential for NMOSD progression, as they strongly correlated with the EDSS score and relapse number in both the discovery and replication cohorts. Notably, the relapse prediction models composed of any combination of key relapse‐related proteins exhibited good performance and similar AUC values. We believe the reason for this is the insufficient number of patients in each cohort.

Plasma exchange (PEX) must be initiated as soon as possible after the diagnosis of acute NMOSD to increase its effectiveness when combined with corticosteroids.^32^ PEX may no longer be the only treatment available because new immunomodulatory treatments, such as complement inhibitors and protease inhibitors, that directly interfere with the effector molecules of NMOSD pathogenesis could become acute‐phase therapeutics. For example, eculizumab is approved only as an interval medication for secondary prevention of sudden exacerbations of NMOSD symptoms. Nonetheless, eculizumab is being used off‐label in patients with NMOSD suffering from severe symptom exacerbations, while PEX provides little to no benefit, as well as during acute episodes of hemolytic‐uremic syndrome and additional diseases linked to acute complement factor 5 (C5) activation.^33^ Therefore, if further research is performed, our selected proteins could serve as significant biomarkers in personalized treatment plans to optimize therapeutic effectiveness and minimize the risks and costs associated with side effects.

Many proteins in our proposed model for predicting NMOSD progression have been confirmed in other studies for their clinical significance in neuroimmune disease (Table S3). Some of these proteins, such as PD‐L1,^34^ MCP‐2,^35^ SLAMF1,^36^ and GDNF,^37^ are recognized as important markers for predicting outcomes and response to treatment, while relatively few reports exist on the association between DNER^38^ or FGF‐23^39^ and CNS disease. The anti‐Tr/DNER antibody is associated with paraneoplastic neurological syndrome (PNS). F. Bernal et al.^38^ demonstrated that there is a strong association between anti‐DNER antibody and the paraneoplastic cerebellar degeneration (PCD) associated with Hodgkin disease (HD). FGF‐23 is a hormone secreted by bone‐embedded osteocytes. In human chronic kidney disease (CKD), elevated levels of FGF‐23 have been shown to be related to adverse health outcomes. These findings also extend to CNS disorders: the overexpression of FGF‐23 may have adverse effects on the brain based on existing in vivo animal models and clinical human studies.^39^, ^40^, ^41^, ^42^ Cell‐surface SLAM family receptors can modulate certain critical immune functions. Immune cell activation can be measured by the induction of SLAM expression. Therefore, changes in SLAMF1 expression can reflect a patient's response to treatment or the severity of disease.^36^, ^43^ GDNF is a neurotrophic growth factor that is important for the survival, maintenance, and regeneration of nerve tissue. Masahiko Yamamoto et al. reported that GDNF and GDNFR‐a mRNA levels were increased to different degrees in the affected nerves.^44^ Notably, in our study, a proportion of patients suffering from severe disease manifestations with acute NMOSD had markedly increased PD‐L1 and MCP‐2 levels, even after receiving corticosteroid or immune‐modulation treatment before the sample was taken. MCP‐2/CCL8 is a chemoattractant that attracts inflammatory cells such as monocytes and is involved in various infectious and inflammatory diseases of the CNS, such as multiple sclerosis (MS) and herpes simplex virus type 1 (HSV‐1) encephalitis (HSE).^35^, ^45^ The PD‐1/PD‐L1 pathway is a crucial immunological checkpoint that plays a vital role in regulating immune system balance and tolerance.^46^ The PD‐1/PD‐L1 pathway inhibits T‐cell function, thereby suppressing the immune response. Therefore, this pathway often reduces disease severity and protects the CNS from immunopathological damage. Previous reports have indicated that the expression of PD‐L1 on microglia increases upon exposure to IFN‐gamma.^47^ In a traumatic brain injury (TBI) model, intrinsic PD‐L1 signaling is crucial for regulating the timing and intensity of astrocyte reactions.^48^ Sheetal Bodhankar et al. demonstrated that blocking the PD‐L1 checkpoint decreased the size of middle cerebral artery occlusion (MCAO) infarctions and enhanced neurological recovery.^49^ This finding suggested that PD‐L1 might play a pathogenic role. The expression of PD‐1 family members in NMOSD has not yet been elucidated in detail. Moreover, the relationships between PD‐L1 and MCP‐2 levels and acute disability scores support the idea of a causal connection between the mobilization and stimulation of cytokines/chemokines, brain tissue injury, and the progression of disability in patients with NMOSD. Many of the indicators evaluated in this study had concentration ranges that overlapped between the acute stages of mild and severe NMOSD, rendering them inadequate for predicting individual exacerbations. Furthermore, the levels of these indicators did not correspond with disability scores, perhaps due to their modulation reflecting downstream effects on the inflammatory response in patients with NMOSD.

This study has several limitations. First, we focused on a risk assessment model comprising inflammatory protein profiles at the acute phase, and dynamic changes in inflammatory biomarkers were not observed. Despite the present findings reflecting the immunological characteristics of NMOSD patients, our study was a single‐center study, and expanding the sample size and conducting prospective verification in multiple centers will be needed in the future. Second, due to the limited patient population, this study included a small number of patients using immunosuppressants, which may have affected the determination of their relapse course. The number of participants in this study was insufficient for multivariable analysis. Third, further investigation of the correlation of the immune interaction between peripheral blood and the CNS is needed. Fourth, additional in vivo and in vitro models to explore the function of protein markers will complement the present findings.

To summarize, we analyzed the inflammatory protein profile of patients in the acute phase of NMOSD, and we screened six clinically relevant proteins. Based on these biomarkers, we constructed prediction models that can accurately differentiate between relapse and attack severity groups with NMOSD. Our findings not only lay the foundation for blood‐based tests for predicting and monitoring the disease course of NMOSD but also provide future directions for research on disease mechanisms and therapeutic targets.

## AUTHOR CONTRIBUTIONS

Quanfeng Wei and Jiahong Li conceived the study, analyzed the data, and drafted the manuscript. The author read and approved the final manuscript.

## FUNDING INFORMATION

This study was supported by the National Key Research and Development Program of China (2021YFA1101403), the Beijing Municipal Public Welfare Development and Reform Pilot Project for Medical Research Institutes (JYY2023‐7), a grant from the Chinese Institutes for Medical Research, Beijing (CX23YZ15), the Project of Construction and Support for High‐level Innovative Teams of Beijing Municipal Institutions (BPHR20220112), the Dengfeng Talent Program (DFL20220701), the Youth Beijing Scholar (NO.020), the Project for Innovation and Development of Beijing Municipal Geriatric Medical Research Center (11000023T000002041657), and the National Natural Science Foundation of China (Grant No. 82101416), and Natural Science Foundation of Liaoning Province (2023JH2/20200149). The funders played no role in the design or conduct of the study; the collection, management, analysis, and interpretation of the data; the preparation, review, or approval of the manuscript; or the decision to submit the manuscript for publication.

## CONFLICT OF INTEREST STATEMENT

The authors declare no conflicts of interest.

## CONSENT TO PARTICIPATE

Participants provided written informed consent to participate in the study before taking part.

## Supporting information


Table S1

Table S2

Table S3

## Data Availability

The datasets used and/or analyzed during the current study are available from the corresponding author upon reasonable request.
