# Supplementary material for: Blood‐based inflammatory protein biomarker panel for the prediction of relapse and severity in patients with neuromyelitis optica spectrum disorder: A prospective cohort study
Source: CNS Neurosci Ther. 2024 Jun 23;30(6):e14811. doi: 10.1111/cns.14811 (PMC11194177; doi:10.1111/cns.14811)
Supplement: Supplementary file 1 — Table S1 [file CNS-30-e14811-s002.docx]

**Table S1** - Clinical characteristics of relapsed patients and non-relapsed NMOSD patients.

|  | Discovery cohort | |  | Validation cohort | |  |
| --- | --- | --- | --- | --- | --- | --- |
|  | Relapse | No relapse | *p* value | Relapse | No relapse | *p* value |
| Patient number, No. | 10 | 20 |  | 9 | 11 |  |
| Female gender, No. (%)  Age, years, mean ± SD | 10(100)  41.30±14.56 | 20(100)  49.85 ±14.83 | *p*>0.999  0.146 | 9(100)  49.22 ±12.98 | 10(90.91)  47.45 ±17.78 | *p*>0.999  0.807 |
| Duration, months (IQR)  Hypertension, No. (%)  Diabetes, No. (%)  Coronary heart disease, No. (%)  Autoimmune diseases, No. (%)  Abnormal rheumatic antibodies, No. (%)  **Clinical manifestation, No. (%)**  ON  TM  ST  MIX  EDSS, median (range)  **Acute attack therapy, No. (%)**  IVMP  IVIG  PE  **Maintenance therapy, No. (%)**  Prednisone(>6months)  MMF  TAC  RTX  CTX  Telitacicept | 44.5 (17.25-87.75)  0(0)  1(10)  0(0)  5(50)  8(80)  3(30)  6(60)  0(0)  1(10)  3.5(3-5.75)  10(100)  0(0)  0(0)  6(60)  4(40)  0(0)  5(50)  1(10)  1(10) | 57.5 (31.75-82.00)  4(20)  6(30)  1(5)  5(25)  12(60)  5(25)  11(55)  1(5)  3(15)  3(3-5)  18(90)  1(5)  1(5)  14(70)  7(35)  1(5)  4(20)  0(0)  0(0) | 0.441  0.272  0.372  *p*>0.999  0.171  0.273  0.869  0.732  0.540  *p*>0.999  *p*>0.999  0.584  0.789  *p*>0.999  0.115  0.333  0.333 | 63 (23.00-65.00)  0(0)  2(22.22)  0(0)  3(33.33)  4(44.44)  5(55.56)  4(44.44)  0(0)  0(0)  6(4-6)  8(88.89)  0(0)  0(0)  8(88.89)  5(55.56)  1(11.11)  1(11.11)  0(0)  0(0) | 40 (27.00-71.00)  0(0)  0(0)  0(0)  4(36.36)  6(54.55)  4(36.36)  3(27.27)  1(9.09)  3(27.27)  2.5(2-4.5)  11(100)  1(9.09)  0(0)  10(90.91)  5(45.45)  0(0)  1(9.09)  0(0)1  0(0) | 0.518  *p*>0.999  0.189  *p*>0.999  *p*>0.999  *p*>0.999  0.251  0.019*  0.450  *p*>0.999  *p*>0.999  *p*>0.999  *p*>0.999  0.450  *p*>0.999  *p*>0.999  *p*>0.999 |

Numerical data are presented as the n (%), mean ± SD, or median (IQR). NMOSD, neuromyelitis optica spectrum disorder; ON, optic neuritis; TM, transverse myelitis; ST, brainstem syndrome; MIX, mixed attack; EDSS, Expanded Disability Status Scale; MMF, mycophenolate mofetil; TAC, tacrolimus; RTX, rituximab; CTX, cyclophosphamide; IVMP, intravenous methylprednisolone; IVIG, intravenous immunoglobulin; PE, plasma exchange.
