# Supplementary material for: Blood‐based inflammatory protein biomarker panel for the prediction of relapse and severity in patients with neuromyelitis optica spectrum disorder: A prospective cohort study
Source: CNS Neurosci Ther. 2024 Jun 23;30(6):e14811. doi: 10.1111/cns.14811 (PMC11194177; doi:10.1111/cns.14811)
Supplement: Supplementary file 2 — Table S2 [file CNS-30-e14811-s003.docx]

**Table S2** The AUC values of the models under different combinations of biomarkers.

|  | Discovery | Validation |
| --- | --- | --- |
| FGF-23+DNER | 0.8100 | 0.8485 |
| FGF-23+GDNF | 0.8600 | 0.8384 |
| FGF-23+SLAMF1 | 0.8400 | 0.8788 |
| DNER+GDNF | 0.7900 | 0.8889 |
| DNER+SLAMF1 | 0.7800 | 0.8788 |
| GDNF+SLAMF1 | 0.7900 | 0.8889 |
| FGF-23+DNER+GDNF | 0.8500 | 0.8687 |
| FGF-23+DNER+SLAMF1 | 0.8450 | 0.8687 |
| FGF-23+GDNF+SLAMF1 | 0.8450 | 0.8687 |
| DNER+GDNF+SLAMF1 | 0.7850 | 0.8889 |

AUC, Area Under Curve
